# Seeking sunlight: rapid phototactic motility of filamentous mat-forming cyanobacteria optimize photosynthesis and enhance carbon burial in Lake Huron’s submerged sinkholes

**DOI:** 10.3389/fmicb.2015.00930

**Published:** 2015-09-07

**Authors:** Bopaiah A. Biddanda, Adam C. McMillan, Stephen A. Long, Michael J. Snider, Anthony D. Weinke

**Affiliations:** Annis Water Resources Institute, Grand Valley State University, MuskegonMI, USA

**Keywords:** cyanobacterial motility, phototaxis, submerged sinkhole, microbial mat, aggregation, dispersion, photosynthetic efficiency, carbon burial

## Abstract

We studied the motility of filamentous mat-forming cyanobacteria consisting primarily of *Oscillatoria*-like cells growing under low-light, low-oxygen, and high-sulfur conditions in Lake Huron’s submerged sinkholes using *in situ* observations, *in vitro* measurements and time-lapse microscopy. Gliding movement of the cyanobacterial trichomes (100–10,000 μm long filaments, composed of cells ∼10 μm wide and ∼3 μm tall) revealed individual as well as group-coordinated motility. When placed in a petri dish and dispersed in ground water from the sinkhole, filaments re-aggregated into defined colonies within minutes, then dispersed again. Speed of individual filaments increased with temperature from ∼50 μm min^-1^ or ∼15 body lengths min^-1^ at 10°C to ∼215 μm min^-1^ or ∼70 body lengths min^-1^ at 35°C – rates that are rapid relative to non-flagellated/ciliated microbes. Filaments exhibited precise and coordinated positive phototaxis toward pinpoints of light and congregated under the light of foil cutouts. Such light-responsive clusters showed an increase in photosynthetic yield – suggesting phototactic motility aids in light acquisition as well as photosynthesis. Once light source was removed, filaments slowly spread out evenly and re-aggregated, demonstrating coordinated movement through inter-filament communication regardless of light. Pebbles and pieces of broken shells placed upon intact mat were quickly covered by vertically motile filaments within hours and became fully buried in the anoxic sediments over 3–4 diurnal cycles – likely facilitating the preservation of falling debris. Coordinated horizontal and vertical filament motility optimize mat cohesion and dynamics, photosynthetic efficiency and sedimentary carbon burial in modern-day sinkhole habitats that resemble the shallow seas in Earth’s early history. Analogous cyanobacterial motility may have played a key role in the oxygenation of the planet by optimizing photosynthesis while favoring carbon burial.

## Introduction

“I then almost always saw, with great wonder……many little living animalcules, very prettily a-moving.”

– Anton van Leeuwenhoek (The Royal Society, 1683)

Over 3 billion years of evolutionary history has endowed cyanobacteria with an arsenal of time-tested structural adaptations and physiological mechanisms enabling them to thrive under environmental extremes (Falkowski et al., 2008; Voorhies et al., 2015). Even today, cyanobacteria often dominate the upper layers of microbial mats found in a variety of extreme environments such as hot springs, hypersaline desert lakes and sub-glacial polar environments (Stahl, 1995; Andersen et al., 2011; Buhring et al., 2011). Motility is one of the many useful life strategies for optimizing microscale gradients of physical and chemical parameters of relevance to microbes living in microenvironments (Stocker, 2012). For filamentous mat-forming cyanobacteria, motility is the key to optimizing environmental conditions for individual organisms within the three-dimensional microbial mat context (Park et al., 2003; Tamulonis et al., 2011). The interweaving of individual trichomes (“filaments,” hereafter) throughout the structure of the mat creates an interlocking web, capable of rapid horizontal as well as vertical movement (Castenholz, 1967; Biddanda et al., 2009). The mat may also be stratified, with different layers of photosynthetic, chemosynthetic, and heterotrophic microbes coexisting in a symbiotic relationship, synchronously moving to obtain their respective resources in cyclic diel patterns (Kruschel and Castenholz, 1998; Nold et al., 2010; Biddanda et al., 2012).

Cell movement is a fundamental but usually unnoticeable feature of the microbial world. In dynamic microbial mat communities, motility is essential to obtain physical resources and maintain the beneficial mat structure (Mitchell and Kogure, 2006). The movement of individual filaments interweaving together and coming into contact creates a mat that can respond to stimuli more efficiently than individual filaments could (Castenholz, 1968). This action is characterized by mass movement of sections of microbial mat driven by the random motility of individual filaments bending, pushing, and gliding by one another (Hoiczyk, 2000). Within this writhing mat, individual cyanobacteria continue moving until coming into an area of favorable physical conditions, wherein they cease movement (Mitchell et al., 1991, 1995). One of the strongest stimuli that the majority of cyanobacteria respond to is light, as they actively sense light gradients and move into or out of discrete photic zones (Dusenbery, 1998). This behavior is responsible for diel migrations within mat communities, wherein cyanobacteria migrate vertically to optimize light conditions (Richardson and Castenholz, 1987; Garcia-Pichel et al., 1994; Kruschel and Castenholz, 1998). The cyanobacteria also respond to discrete chemical signals, exhibiting chemotactic movement to optimize chemical intake or avoid harmful environments (Chet and Mitchell, 1976). Such dynamic behavior appears to contribute to the unique microbial mat structure and its interactions with the environment.

**FIGURE 1:**
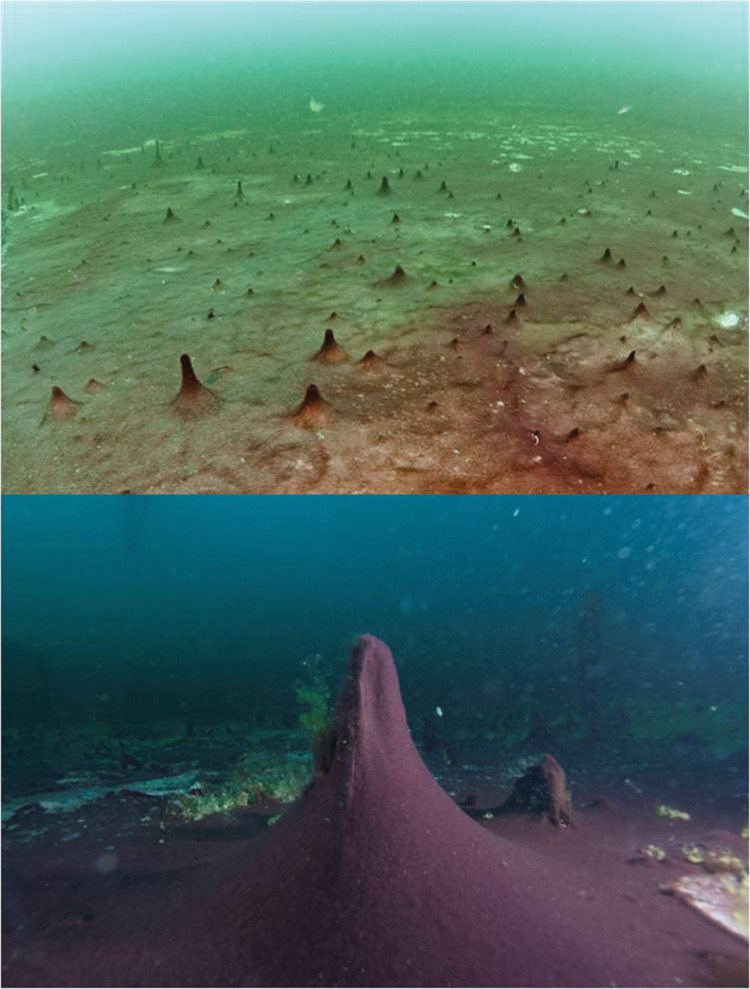
**Diver photos: underwater panoramic imagery of cyanobacterial mat covering the lake floor at the Middle Island Sinkhole, Lake Huron (**top**; horizontal foreground scale = 3 m, background scale = 30 m; photo credit: Tane Casserly, NOAA), and close-up photo of a very large conical mat finger rising due to gas-filled buoyancy (**bottom**; horizontal foreground scale 1m, vertical foreground scale 0.5 m; photo credit: Joe Hoyt, NOAA)**.

Time, water, and geologic forces have converged to create underwater sinkholes in Lake Huron supporting prolific microbial mats that resemble life as it may have existed throughout much of Earth’s deep history (Biddanda et al., 2012). Several such ecosystems can be found in the shallow coastal waters of Lake Huron, close to Alpena, MI – a region underlain by karstic limestone wherein groundwater seeps through Paleozoic marine evaporites (Biddanda et al., 2009, 2012). Two locations, the El Cajon Bay (ECB; 0.25–2 m, with mats receiving 50–90% of sunlight incident at the lake surface) and Middle Island Sinkhole (MIS; ∼23 m, with mats receiving 5–10% of sunlight incident at the lake surface), contain groundwater vents providing habitat for mat-forming cyanobacteria mainly comprised of the genus *Oscillatoria* and *Phormidium* (Voorhies et al., 2012). The venting groundwater is characterized by a relatively constant low temperature (7–9°C), low pH (7.1), low dissolved organic carbon (<1 mg l^-1^), low oxygen (0–2 mg l^-1^), high dissolved inorganic carbon (>40 mg l^-1^) and high sulfate (>1000 mg l^-1^) compared to overlying Lake Huron water (Ruberg et al., 2008; Biddanda et al., 2009). Except for the daily and seasonally variable light climate, the relatively dense groundwater of constant composition that flows over the sediment provides a stable benthic environment. Here, strikingly purple mats formed by filamentous mat-forming cyanobacteria capable of oxygenic and anoxygenic photosynthesis, cover the bottom sediment (Biddanda et al., 2012; Voorhies et al., 2012; **Figure 1**). These cyanobacteria are closely related to those inhabiting the bottom of certain Antarctic lakes and hot springs (Castenholz, 1968; Hawes et al., 2011, 2013; Voorhies et al., 2012), and such ecosystems represent analogs to the most ancient forms of life on Earth (Allwood et al., 2006). Metagenomic studies of cyanobacterial benthic microbial mat communities in Antarctic lakes indicate that the consortia of benthic microorganisms are capable of nutrient scavenging and recycling, allowing the proliferation of large densities of organisms despite nutrient-poor surroundings (Andersen et al., 2011). In Lake Huron’s submerged sinkholes, the photosynthetic filamentous cyanobacteria form a mat together with white, chemosynthetic bacteria in stratified layers (Biddanda et al., 2009; Nold et al., 2010). In *Oscillatoria*, translocation is often accompanied by species-specific rotations around the long axis of the filaments (Hoiczyk, 2000). Given the motility of *Oscillatoria*, we consider that it serves to enhance the growth and survival of the microbial mat community by enabling better exploitation of the resource base in their low-oxygen, high-sulfur benthic environment. Ability of filaments to move at environmentally meaningful rates may confer major advantages to *Oscillatoria* for closely tracking and acquiring light and limiting nutrients across the diurnally variable light and sharp redox gradients that prevail in submerged sinkhole ecosystems. If confirmed, such a motile life strategy by mat-forming cyanobacteria should have significant collective impact on the biogeochemistry of these lacustrine benthic habitats.

We used bright-field microscopy and image analysis software to analyze individual and group motility of sinkhole mats primarily composed of filamentous *Oscillatoria* in microcosms filled with freshly collected ground water from the sinkholes. Photosynthetic yield was measured to quantify the cellular benefit of phototaxis. We also examined the motile behavior of the microbial mat to different stimuli, such as temperature fluctuations that are likely to be prevalent in the environment. Lastly, we utilized intact sediment cores with overlying mats bathed in groundwater to observe filament motility and track particulate matter burial in slow motion.

## Materials and Methods

### Groundwater and Mat Collection, and Storage

Following a detailed water-column profile at the sinkholes using YSI sondes equipped with sensors for temperature, pH, conductivity and dissolved oxygen, groundwater was collected by closing Niskin bottles below the depth of the thermo-chemocline and as close to the bottom of the lake as possible without disturbing the sediment (usually at about 0.5–1.0 m above the mats). Collected groundwater was stored refrigerated in the dark until its use as media in mat motility experiments. Microbial mat samples containing mostly *Oscillatoria*-type cyanobacteria (Voorhies et al., 2012; *Oscillatoria* herein) were collected in the ECB and at the MIS (Nold et al., 2010). At the shallow ECB, samples were collected by hand from a kayak over the shallow springs. At the deeper MIS, divers collected the samples by hand in cores with rubber stoppers at either end. The samples were stored in groundwater on ice in a cooler until return to the laboratory (2–5 h), and were then stored in a refrigerator. All mat samples were used for experiments within 1–2 weeks of their collection.

### Aggregation and Dispersion

For macroscopic aggregation and dispersion observation, *Oscillatoria* mat samples were placed into plastic Petri dishes (8.5 cm diameter wide; 1.5 cm deep) in room temperature groundwater and motile behavior was observed. Cyanobacteria were let to sit for motile behavior to be observed. Images were taken using a digital camera (SeaLife DC1200). For microscopic dispersion observation, already formed colonies of cyanobacteria were placed under a dissecting microscope (NikonSMZ-27) set at 10X magnification. Time-lapse images were taken at 2.5 min intervals using a microscope camera (Nikon Publisher 5.0 RTV QImaging Camera) and processed using software (Nikon QImaging Software). High magnification images were obtained by a Nikon eclipse 80i microscope using NIS elements basic Research Software, and photograpahed with a QIClick digital camera (QImaging, Surry, BC, Canada) as described in Voorhies et al. (2012).

### Temperature-Dependent Motility

Temperature-dependent motility of individual filaments was observed through a dissecting microscope at ∼6X magnification. A circle of laminated graph paper with grids of 1 mm^2^ dimension was taped with electrical tape to the bottom of a plastic Petri dish. The Petri dish was then taped to the bottom of a wide, shallow glass bowl. The entire apparatus was placed under the dissecting microscope. The Petri dish was filled with groundwater at 10°C and forceps were used to place a small colony of *Oscillatoria* into the dish. The individual filaments were agitated in the Petri dish to an approximately even density, and then allowed to settle to the bottom of the dish. The outer glass bowl was filled with groundwater of the desired temperature for the experiment. Groundwater was taken directly out of the fridge and immediately placed on a hot plate until the correct temperature was reached. The warmed up groundwater was then poured into the shallow glass bowl and allowed to surround the Petri dish. The temperature of the groundwater in the Petri dish was monitored by two digital thermometers every 5 min. A relatively constant temperature of groundwater was maintained in the Petri dish by siphoning out the water in the surrounding bowl and replacing it with water of the intended temperature after each reading, and the system worked as an effective thermostat. The motility of the *Oscillatoria*, was monitored under the dissecting microscope over the 1 mm^2^ gridded graph paper. Time-lapse images of the motility of individual filaments were taken at 2-min intervals for an hour. Speed measurements were calculated using image analysis software (QImaging Software).

### Measurement of Phototaxis

Phototaxis was observed to determine the precision of the response of *Oscillatoria* to light. The edge of a plastic 8.5 cm diameter Petri dish was covered with opaque electrical tape. The top of the Petri dish was covered in aluminum foil with the cutout of a specific symbol. The Petri dish was filled with groundwater and cyanobacteria were allowed to sit for half of a day, first aggregating and then dispersing. The cyanobacteria were spread out evenly throughout the Petri dish as a mat once the experiment began. The distance between the top of the Petri dish covered by foil and the bottom where mat filaments settled down was ∼1.5 cm. The aluminum covered top of the Petri dish was put on the top of the dish and placed under a lamp of 75 μmol photons m^-2^ s^-1^. The mat sample in the Petri dish was left under the lamp at a distance of ∼25 cm for 1–2 h. The results of the experiment were recorded using a digital camera.

Time-lapse photography of *Oscillatoria* exhibiting phototaxis was taken under a dissecting microscope (NikonSMZ-27) using a microscope camera (Nikon Publisher 5.0 RTV QImaging Camera). For certain experiments (**Table 1**, A and B) the lens to the stage was covered in an aluminum foil dome of ∼10 cm dimensions to prevent room light from intruding. A light source with a bendable arm was covered with a plastic pipette (disposable polyethylene transfer pipette of narrow shape and 1 ml capacity) by cutting the bulb of the pipette in two and placing the severed bulb over the head of the light source. The plastic pipette was also covered in aluminum foil, reducing the beam of light to a pinpoint. An ∼1.5 cm diameter hole was cut into the aluminum foil surrounding the stage of the microscope and the light source pipette threaded through the hole, allowing the pinprick of light to point into the Petri dish. The hole was sealed with opaque, black electrical tape with the pipette pierced through the hole so as not to allow any excess room light into the aluminum dome. The pipette was threaded into the hole so that the light source was flush with the aluminum dome and the pipette tip (∼2 mm diameter) was only millimeters from the surface of the groundwater. In this configuration the light traveled from the light source down the length of the pipette (∼12 cm) before entering the groundwater containing the filaments. Another hole was cut into the aluminum foil and covered by a removable aluminum foil flap taped to the shell. A Petri dish with *Oscillatoria* was placed on the stage and the aluminum foil completely restricted room light from reaching the stage. Time-lapse photography was started to observe the effect of the pinprick of light on the cyanobacteria. Seconds before a picture was taken, the flap in the aluminum foil dome was removed, allowing room light to hit the experiment and illuminating the cyanobacteria, allowing an accurate picture to be taken. Immediately after the picture was taken, the removable flap was replaced and the experiment continued.

**Table 1:** Compilation of permalink You-Tube clips showing short time-lapse photography sequences of several *Oscillatoria* filament and mat motility events.

Description of motility event	Time-lapse video Youtube Permalink
(A) Filaments showing positive phototaxis towards a pin point of light.	https://youtu.be/14qzO9sh8Fc
(B) Filaments showing positive phototaxis towards a beam of light.	https://youtu.be/UGAQ91tEbqs
(C) Time-lapse movement of filaments over a 1mm graph paper background.	https://youtu.be/VUACHE3RoG4
(D) Filaments showing movement across a boundary zone.	https://youtu.be/mSjJWEO-cVk
(E) Gradual filament movements covering and burying a tiny pebble.	https://youtu.be/9TnbuM1Rxwg
(F) Rapid vertical and horizontal motility covering up a large pebble.	https://youtu.be/syfmu8SVZE8

*(A) *Oscillatoria* from El Cajon exhibiting phototaxis toward a pinpoint of light, then spreading out when the light is turned off. (B) *Oscillatoria* from El Cajon exhibiting phototaxis toward a beam of light. (C) The linear motility of single filaments of *Oscillatoria* over 1 mm × 1 mm gridded graph paper, used to find speed. (D) The border of a clump of *Oscillatoria* that had been recently placed into a petri dish spreading out over 1 mm × 1 mm graph paper. (E) The burial of a small pebble beneath a mat of motile cyanobacteria. (F) Rapid climbing behavior of filaments over a small piece of pebble placed on top of the mat completely covering it within hours.*

### Effect of Phototaxis on Photosynthetic Yield

Photosynthetic yield (Fv’/Fm’, a ratio of fluorescence) of PSII, the oxygen evolving photosynthetic complex, was measured with pulse amplitude modulation (PAM) with a Walz DIVING-PAM submersible fluorometer (Walz, Germany). This instrument measures back-fluorescence of photosystem II (PSII), and can be roughly understood as an indicator of photosynthetic health (Campbell et al., 1998). Filaments were placed into a plastic Petri dish full of groundwater at room temperature and allowed to sit. After aggregating and then dispersing, the cyanobacteria formed a mat. The top of the Petri dish was covered in aluminum foil and four small holes were cut in the foil. The Petri dish was placed under a lamp of 75 μmol photons m^-2^ s^-1^. The cyanobacteria were allowed to migrate for 1 h. At 10 min intervals, the lamp were shut off and the foil cover taken off of the Petri dish. At this time, the Diving PAM was used to measure the photosynthetic yield of the four small colonies that had migrated into the light and four places that had been dark and under the foil. The top of the Petri dish was then replaced and the lamp turned back on.

### Burial Experiment

The cyanobacterial mat from the ECB was carefully placed into a plastic core of sediment (7 cm diameter) and bathed in groundwater from the site. An intact mat grew and covered the sediment over 1 day, with artificial lights on during the day and lights off at night. Small pieces of bivalve shells and pebbles of unknown origin collected from the local Lake Huron beach (0.3–0.8 mm) were rinsed with the ground water and carefully placed one at a time upon the mat. Over a period of 3 days the artificial diurnal cycle was continued and digital images were taken of the mat and shells or pebbles multiple times a day. The experiment ran for a total of 3 days, with pictures being taken every hour of the day for those 3 days.

## Results

### Aggregation and Dispersion

**FIGURE 2:**
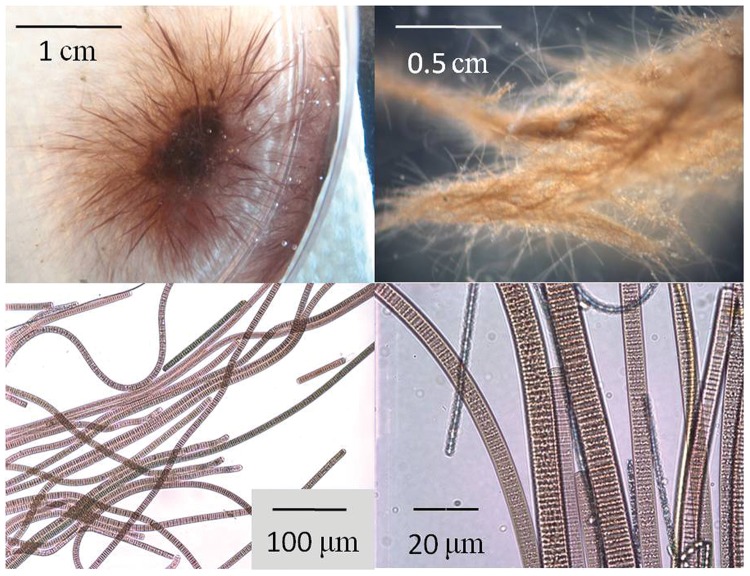
**Microscopic images of *Oscillatoria* filaments collected from benthic mats at El Cajon Bay and Middle Island Sinkhole, Lake Huron. Top left**: Low magnification digital camera image of the dispersion of individual filaments form a recently formed aggregation of Oscillatoria in a petri dish; **Top right**: low magnification microscopic image showing Oscillatoria filaments in a freshly collected mat sample from Middle Island Sinkhole; **Bottom left:** medium magnification microscopic image of *Oscillatoria* filaments; **Bottom right:** high magnification microscopic image of *Oscillatoria* filaments, with pigmented smaller filaments likely representing the cyanobacteria *Phormidium*, and the non-pigmented smallest filaments likely to be chemosysnthetic bacteria or archea.

**FIGURE 3:**
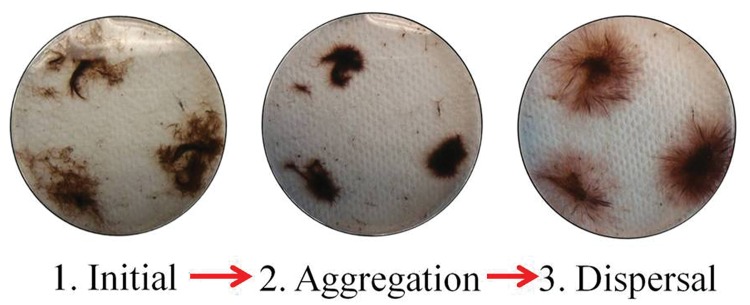
**Sequence of events starting with initial aggregation ending with dispersion of *Oscillatoria* from Lake Huron sinkhole mats placed in ground water in a 8.5 cm diameter plastic Petri dish at 23°C photographed at ∼10-min intervals**.

**FIGURE 4:**
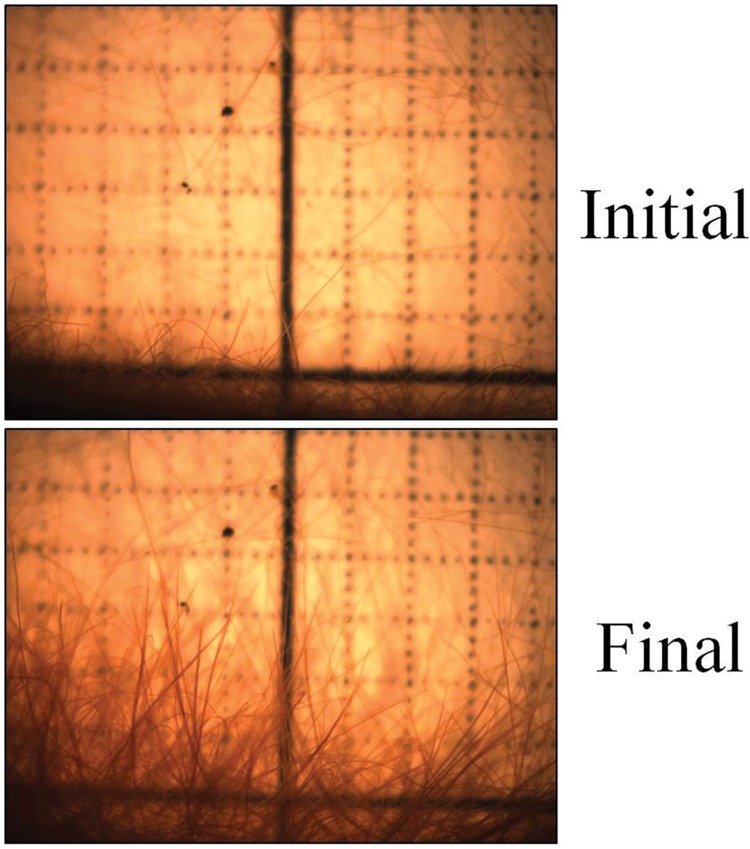
**Filament movement across a boundary zone.** Dispersion of filaments and tendrils of interwoven filaments along the periphery of a colony in ∼25°C groundwater in a plastic Petri dish placed over 1 mm^2^ gridded graph paper. The second image was taken after 50 min.

The *Oscillatoria* from Lake Huron showed a consistent behavioral pattern upon being placed into a new environment. When placed into a still groundwater medium and agitated briefly with a probe such as a pipette tip, the filaments collected into density dependent colonies (**Figure 2**). If the density of filaments was high, one single colony formed, and if the overall density of filaments was low, a number of smaller colonies of high density formed (**Figure 3**). These colonies are not easily separated by agitation of the medium, but can be separated by mechanical means with some persistence. In room temperature groundwater, aggregation takes about 20 min for 1–2 weeks old *Oscillatoria*. Following aggregation, the high-density colonies “sent out” tendrils of interwoven filaments around the periphery of the colony (**Figure 4**). These tendrils separated into individual filaments as distance from the colony increased, eventually resulting in an even spread of filaments throughout the medium. If density was high enough, filament colonies first came into contact by colony “bridges” interconnecting two or more colonies. These sub-colonies then aggregated into one central colony.

### Temperature-Dependent Response

**FIGURE 5:**
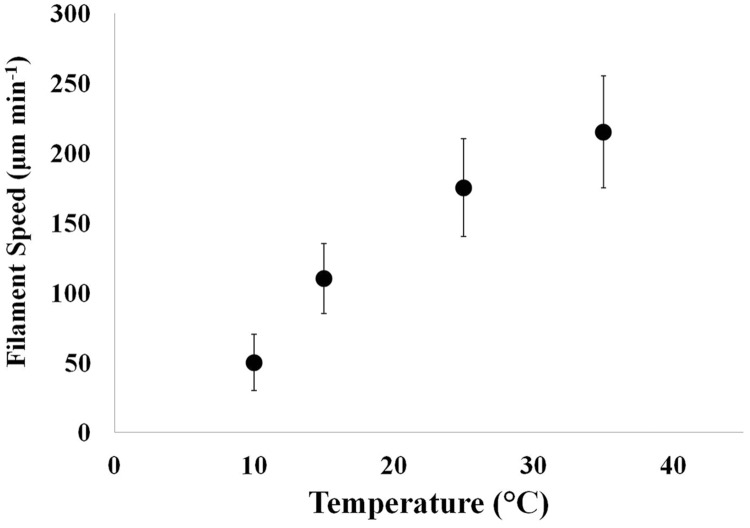
**Change in filament motility in response to changes in water temperature.** As temperature increased in the groundwater medium, motility speed of single filaments increased. The temperature range tested was 10–35°C. Microscopic images of single filaments in a plastic Petri dish containing groundwater placed over 1 mm^2^ graph paper at 10X magnification were captured at 2-min intervals and analyzed using image software analysis. Error bars represent 1 SD over *n* = 44–174 individual measurements of filament speed.

Single filaments of *Oscillatoria* showed increased speed with increasing temperature of the groundwater medium (**Figure 5**). Filaments survived in the range of 4–45°C. At the upper temperature range limit, filaments quickly turned green and ceased motility. It is quite possible (but unknown at the present time) that at very high temperatures, the pycobiliproteins denature before chlorophyll – causing a color change from purple to green.

### Phototaxis

**FIGURE 6:**
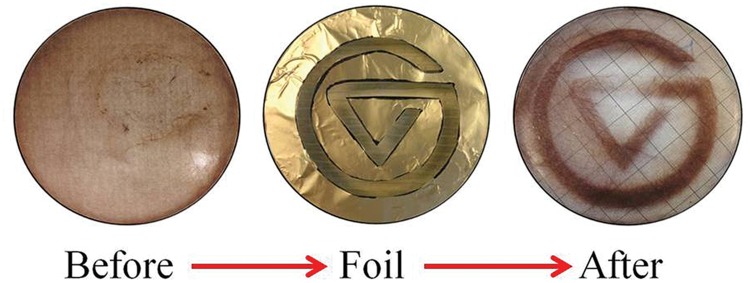
**Filament phototaxis.** The filaments in a thin mat layer respond quickly to a light stimulus by precisely coordinating their position in the zone of light. The logo of Grand Valley State University was cut out of foil and placed over a 8.5 cm diameter plastic Petri dish cover, which was then placed over the plastic Petri dish. The before and after images, were taken 30 min apart.

Filaments of *Oscillatoria* exhibited positive phototaxis. This positive phototactic response was found to be extremely precise, coordinated, and occurred at a measurably rapid pace. The filaments in a microbial mat were quickly able to move into the area of light shining through a foil cutout design and align themselves precisely within the zone of light (**Figure 6**). A single beam of light and a pinpoint of light shining on the mat showed the same effect under the microscope. While the light was shining on the mat, the filaments exhibited positive phototaxis toward the light and aligned precisely within the shape of the light. When the light was turned off, the filaments dispersed back into the mat and spread out to their original formation (**Table 1**, A and B). These positive phototactic responses and the spreading out when the light is turned off both took around 20 min. Filament motility appears to occur smoothly and over a longer period of time. The video links in **Table 1**(A and B) show the positive phototaxis, and those in **Table 1** (C and F) show the other varying movements. A search of the literature revealed that these *Oscillatoria* with a speed of roughly 50 μm min^-1^, were in particular quite rapid for gliding bacteria, and in general faster than other non-flagellated/ciliated prokaryotic microbes (Milo and Phillips, 2014). However, the *Oscillatori* in the present study were slower than flagellated/ciliated prokaryotes and larger motile eukaryotic microbes in terms of absolute and as well as relative speed (**Table 2**). *Oscillatoria* outpace *Listeria* and *Myxococcus*, but are much slower than *Salmonella* and *Escherichia*.

**Table 2:** Comparison of linear distance traveled per unit time (speed) and number of body lengths traveled per unit time (relative speed) for various prokaryotic microbial species and select eukaryotes.

Organism	Absolute speed	Relative speed	Reference^∗^
(Genus/common name)	(μm min^-1^)	(Body Lengths min^-1^)	
**Prokaryotes**			
*Listeria monocytogenes*	6	3	Milo and Phillips (2014)
*Myxococcus xanthus*	10	5	Milo and Phillips (2014)
*Oscillatoria*	50	16	Present Study
*Beggiatoa*	120	–	Encyclopedia of Science and Technology [EST] (1960)
*Eschrichia coli*	900	450	Milo and Phillips (2014)
*Salmonella*	1200	–	Encyclopedia of Science and Technology [EST] (1960)
*Spirillum*	3000	–	Encyclopedia of Science and Technology [EST] (1960)
*Vibrio*	4500	2250	Encyclopedia of Science and Technology [EST] (1960)
*Bdellovibrio*	9600	4800	Milo and Phillips (2014)
**Eukaryotes**			
Copepod	6.0 × 10^5^	600	Mazzocchi and Paffenhofer (1999)
Human (Swimming)	1.0 × 10^8^	50	Milo and Phillips (2014)
Human (Sprinting)	5.5 × 10^8^	360	Cavagna et al. (1971)
Cheetah	1.5 × 10^9^	750	Wilson et al. (2013)

*^∗^Full details under References. Milo and Phillips (2014) = Cell Biology by Numbers, Weizmann Institute of Science, Encyclopedia of Science and Technology [EST] (1960) = McGraw Hill, and Mazzocchi and Paffenhofer (1999) = Swimming in copepods.*

### Photosynthetic Yield

**FIGURE 7:**
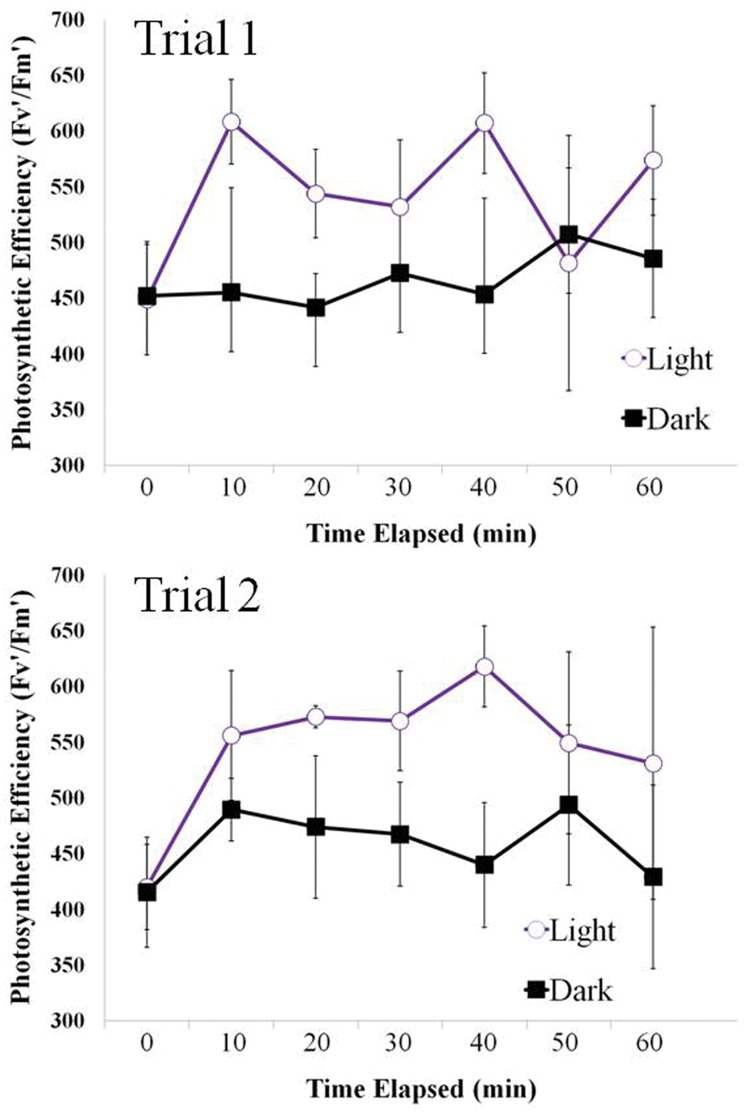
**Changes in photosynthetic efficiency over time in Light and Dark treatments measured at 10 min intervals.** Photosynthetic efficiency (Fv’/Fm’ ratio of fluorescence, is unit-less and usually expressed in the scale of 0–1, or in an expanded range of 0–1000, where 500 represents 0.5 in the original scale – as in the present study) was elevated in the lighted areas under circular areas of foil-cutouts where filaments had aggregated in both trials runs.

Similarly to the positive phototaxis resulting in a symbol created by motile filaments above, the filaments showed the same response with a foil cutout of four different small holes. The filaments in the light zone were found to have a higher photosynthetic yield than filaments in the dark (**Figure 7**), indicating increased photosynthetic advantage in these areas. There was a positive correlation between positive phototaxis and photosynthetic yield.

### Burial

**FIGURE 8:**
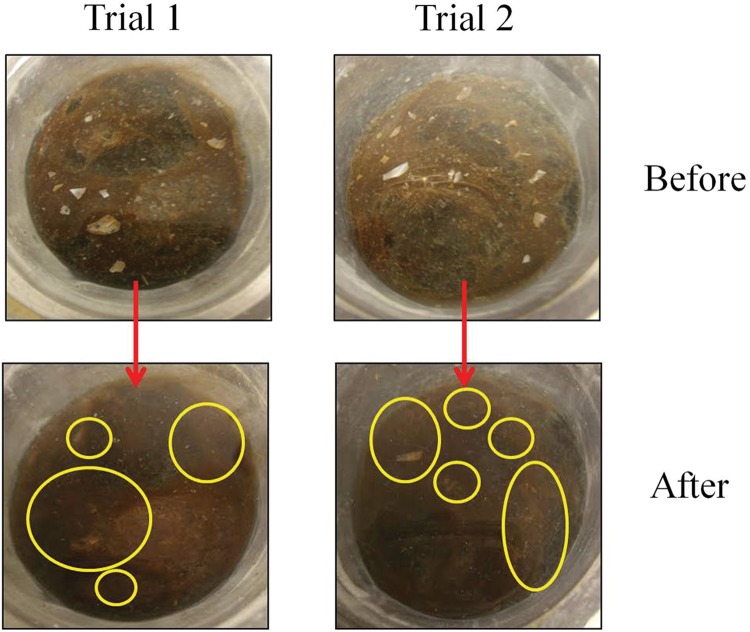
**Observations of particulate matter burial.** Pieces of shells placed on an intact mat became completely buried in the sediment underneath due to the mat over-growth in about 3 days. The mat was grown in groundwater at room temperature overlaying sediment cores taken from the site of mat sampling. Circles point our attention to sites where shells were visible on top of the mat (before), and the same sites where they have disappeared under the mat (after).

Small pieces of shells that were placed upon an intact microbial mat were entirely buried over a period of 3 days (**Figure 8**). The mat was interwoven with enough strength to peel it away as a solid layer and the shells were found beneath the mat in the same locations in a horizontal plane. A similar result occurred with small pebbles (∼0.3–0.8 dimension) placed upon a mat. Time-lapse photography showed that the motile single filaments completely covered the pebble in a thin layer of mat that was ∼20 μm thick over just a period of an hour or two (**Table 1**, E and F).

## Discussion

### Coordinated Motility of Sinkhole *Oscillatoria* Optimize Mat Building and Light Acquisition, and Result in Efficient Carbon Burial

Throughout Earth’s history, tiny organisms that composed massive colonial mats, such as the lithifying stromatolites and other non-lithifying cyanobacterial mats, have left big foot prints either in the geological record or the composition of the air and water, or both (Falkowski et al., 2008; Kamp et al., 2008). It is generally accepted that cyanobacteria mediated Earth’s oxygenation by their oxygenic photosynthesis, and it is argued that they may have prolonged the low-oxygen period in Earth’s history by simultaneously practicing anoxygenic photosynthesis (Voorhies et al., 2012). Despite the long period of cyanobacterial and Earth’s co-evolution that took place when oxygen was low (>1 billion years), and the critical geobiological turning points that occurred under those conditions, very little is known about the physiology and life styles of cyanobacteria that thrive under conditions of low-oxygen and high-sulfur (Lyons et al., 2014). Fortunately, modern cyanobacteria exhibit a range of carbon and sulfur physiologies such as respiration, oxygenic photosynthesis, anoxygenic photosyntheis, and chemosyntheis (Falkowski et al., 2008; Buhring et al., 2011), providing a window into our biosphere’s past. Lake Huron’s submerged sinkhole ecosystems are good examples of low-O_2_ cyanobacterial mats where oxygenic primary production driven by inorganic carbon is supplemented by anoxygenic photosynthesis driven by sulfide (Voorhies et al., 2012).

The majority of microbial mats on Earth are still dominated by cyanobacteria, and in these extreme habitats where eukaryotic photosynthetic algae cannot thrive, cyanobacteria serve as the base of the food web (Stahl, 1995). Because microbial mats are characterized by steep redox changes and fluctuating physico-chemical microscale gradients of light, dissolved oxygen and nutrients (Buhring et al., 2011), mat-dwelling microbes exhibit considerable ability to move across these gradients (Ramsing et al., 1997, 2000; Teske et al., 1998; Tamulonis et al., 2011; Stocker, 2012). It is quite conceivable that motility confers significant advantages for optimizing variable life styles of oxygenic photosynthesis, anoxygenic photosynthesis, and chemosynthesis across sharp gradients of resources and physico-chemical conditions that prevail in such low-oxygen and high-sulfur ecosystems (Shepard and Sumner, 2010; Buhring et al., 2011; Biddanda et al., 2012; Voorhies et al., 2012). Compared to the overall motility of other microbes (both in absolute speed and in relative terms such as body lengths per unit time), the cyanobacteria from Lake Huron’s sinkholes occupy a niche at the slower end of the range. However, their motility rates are on the high end of non-flagellated and non-ciliated microbes, and at the lower end of motility rates for flagellated and ciliated microbes (Milo and Phillips, 2014). Perhaps, the cyanobacteria in Lake Huron’s sinkholes living in a relatively cool and steady environment are motile to just the right degree. Their relatively slow and steady life style may be quite adequate for operating at spatial scales relevant to them enabling to form and maintain mat structure, find sunlight and optimize photosynthesis, and in the process, efficiently bury carbon in the underlying sediments – all features that may have been important for oxygenation of early Earth.

### Aggregation-Dispersal and Photosynthetic Yield

Microbes such as those found in and on biofilms not only seek nutrients and light, but also seek out each other by clustering together in coordinated motion. The *Oscillatoria* from ECB and MIS are not only phototactic, having the ability to position themselves precisely within optimal light conditions – but also are capable of active aggregation and dispersal. The aggregation effect of the *Oscillatoria* filaments to create an interwoven mat in the lab is almost identical to the aggregation effect seen in similar thermophilic *Oscillatoria* (Castenholz, 1967). The hallmark of the response is to quickly create mat-like structures of individual filaments dependent upon initial filament density. High-density filaments spread throughout a groundwater medium and form into a singular, large mat. Low-density filaments form into small mat-like clumps in areas of the highest density, resulting in multiple small clumps throughout one Petri dish (**Figure 3**). Our results match closely with studies of mat behavior in other ecosystems, wherein gliding speeds of 1–3 μm s^-1^ (60–180 μm min^-1^) in 1–2 mm long filaments are commonly recorded (Kamp et al., 2008; Tamulonis et al., 2011, **Table 2**). However, the motility pattern continued in the MIS and ECB *Oscillatoria* with a dispersion effect. The individual filaments traveled away from the main colony in a uniform dispersion, staying in contact with the central colony through long tendrils of interwoven filaments. The eventual result was uniform dispersion throughout the medium. This behavior was observed both in the light and in the dark (although at a much reduced speed relative to their speed in the light), suggesting this behavior is not limited to phototaxis, and chemotaxis may be a potential factor. Additional research is needed to identify the ecological role of this motile response in the dark.

Clumping of filaments allows cells to ‘sense’ light over distances far greater than their own cell lengths. This is crucial as random movements at their speed alone may not cause a cell to move into an area that would increase their light optimization (either from an area of low light to high light, or vice versa). Simulated random movement of filaments of increasing length may have a far better chance of finding a more favorable area (Tamulonis et al., 2011). Light is a critical factor for these cyanobacteria, so optimization of light harvesting is vital. Light conditions that are too low will lead to insufficient carbon fixation, and light conditions that are too high are inhibitory and can cause severe cell damage. Ecologically and physiologically meaningful rates of movement are important for responding to rapid changes in light and redox conditions that are likely to be presenting *in situ*. Our results show not only that they are capable of coordinated movement, but also that this movement confers an important biological advantage (seen as an increase in photosynthetic yield), and that they remain in these areas of light (**Figures 6** and **7**). Clustering together as a mat creates other significant biological advantages, such as protection from predation, and even sensing redox gradients (Stocker, 2012).

The strategy of clumping and dispersing over the lake sediment surface all the while staying connected to each other, could be an advantageous tactic for staying put in an ideal environment. If we assume that the early Earth’s shallow seas were turbulent, then mat formation over the benthic environment may have been an advantageous strategy for avoiding being ripped off the sediment and transported to another location that was a less favorable habitat. This strategy may be left over from the past and today prevents them from being transported by the strong storms and wind-induced turbulence of the Great Lakes. By keeping a tight network among the mat, larger sections are more likely to be ripped off the sediment, thus transporting a larger colony that would have a higher chance of surviving in a new environment. The quick motility also would allow those to grow into any new gaps and prevent the remainder of the mat community from being sloughed off in a similar fashion.

The formation of characteristic finger-like projections of mats is caused by the buoyancy of trapped microbially generated gasses such as H_2_S and CH_4_ (Nold et al., 2010, 2013), and appears to be unrelated to motility of filaments seeking light (**Figure 1**). Andersen et al. (2011) analyzed this idea in a similar ecosystem with mat forming cyanobacteria that form similar structures (cones and pinnacles) in the permanently ice-covered lakes in the Dry Valleys of Antarctica. They concluded that the difference in illumination would not likely be different enough between prostrate and raised mats to confer any sort of advantage. Other Antarctic researchers have found that motility is critical for optimizing available resources in these sub-glacial lake environments, and have argued that this feature likely plays a key role in acclimating to environmental changes (Hawes et al., 2011, 2013).

### Temperature and Motility Relationship

The observed increase in speed due to high temperature groundwater could be a reaction to non-ideal ambient physical or chemical conditions. Such behavior has been observed in other mat-forming cyanobacteria as a reaction to multiple stimuli, including depleting nutrient concentrations and damaging UV light intensity (Tamulonis et al., 2011). The ability of the *Oscillatoria* to continue motility in such a wide range of temperature from 10–35°C suggests that the organism has a wide range of temperature tolerance. Additionally, the greater movement at higher temperatures may reflect higher metabolic activity or the advantage for more rapid movement to enable cells to escape an unfavorable micro-environment (Nadeau and Castenholz, 2000).

It is quite possible that the sinkhole mats of Lake Huron occassionally see temperature fluctuations beyond their near-steady state groundwater temperature of 7–9°C. Even the shallow 1–1.5 m deep El Cajon Springs never freeze during the winter due to the continual flux of ∼9°C groundwater. Time-series monitoring using sondes deployed in the bottom of MIS during major storm events has revealed that such events during summer months can occasionally mix warm surface lake water down to 23 m to the mats (Ruberg et al., 2008). Our temperature-motile stimuli response studies show that the *Oscillatoria* can survive in a wide range of temperatures, suggesting this feature could be important in the dispersal and colonization of these cyanobacteria throughout the Karst coastline of Lake Huron, as they are found in multiple sites that have no direct groundwater connection.

It is also possible that the increase in ambient water temperature simply increases the rate of reactions related to motility (Neidhardt et al., 1990). At their ambient low temperatures of 7–9°C, the sinkhole cyanobacteria may be moving as fast as they can when aggregating or dispersing to find a better habitat. In contrast, perhaps they are not moving as fast as they can, but are conserving energy and diverting it to other, more important cellular processes. As conditions become less ideal, such as with increasing temperatures, they may allocate more energy toward motility that would allow them to move faster and find a more suitable habitat. It could be that over their long geobiologic history, cyanobacteria have evolved ecologically advantageous features such as motility that allow them to succeed in sharply contrasting redox environments in which they frequently occur.

Many aspects of cyanobacterial motility remain unexplored. Motility that has been observed in the present study has been primarily phototactic and chemotactic. Although the precise mechanisms of cyanobacterial motility are still unknown, earlier studies have revealed certain structural and physiological key features such as: slime secretion processes, organelles, and distinct surface proteins (Hoiczyk, 2000). With such precise photomovement capabilities, specific mechanisms for motility presumably exist, and further *in vitro* microscopic research will be needed to reveal them.

### Phototaxis

Gliding of cyanobacteria is controlled by a number of external stimuli, but when residing in a calm environment, light seems to be the most important (Hoiczyk, 2000). Others have found that in addition to phototactic movement, the long filament shape also serves to maximize light exposure of the cells (Tamulonis et al., 2011). Phototactic positioning in an ideal microenvironment works best for long filamentous bacteria that are in a microbial mat as they can indirectly sense light many lengths greater than their individual cell lengths.

The interweaving of the filaments in a mat formation in the *Oscillatoria* allowed for the precise phototactic response seen in **Figure 4**. Further, the increase in photosynthetic yield remained elevated throughout the duration of the experiment, indicating that the cells remained in the favorable photosynthetic conditions. This particular experiment looked at horizontal movement in response to light, a phenomenon that is more relevant to ECB than MIS. The deep benthic environment of MIS is relatively flat and light is distributed equally to all parts, whereas the shallow benthic environment of ECB has significant macrophyte vegetation and floating algal mats that may cause small areas of shade resulting in inhomogeneous light distribution. The depth of the water column above the microbial mat at the MIS site filters the majority of light before it reaches the mat (∼95%), allowing only low intensity light to reach the lake floor. Vertical phototaxis (discussed below) may allow for the *Oscillatoria* to remain on top of the mat during the day, as many similar cyanobacteria exhibit negative phototaxis when in direct light. Mat-forming cyanobacteria, *Microcoleus chthonoplastes* found in hypersaline mats at Salins-de-Giraud, France, exhibit phototactic migrations to the upper layer of the mat during the day and are spread homogenously through the mat at night (Tamulonis et al., 2011).

During the night, the white chemosynthetic bacteria are present at the top of the mat, and the *Oscillatorians* rest underneath. It is unknown whether the chemosynthesizers move toward the surface, whether the *Oscillatorians* descend in this diurnal cycle, or if a combination of both occurs. However, if the filaments are descending, it would be a motile response that is not phototactic – but is likely to be a chemotactic response toward fluctuating redox at night when oxygen production ceases. At the ECB, the *Oscillatoria* are found upon, under, and around plant growth, detritus and other stationary objects within the water saturation of the groundwater spring. These features allow for the cyanobacteria to migrate to the shade if needed. *Oscillatoria*, having longer filaments that allow them to measure light between more distant points, which enhances their capacity to navigate with noisy light signals, detect subtle gradients and avoid falling into small sub-optimal light traps (Tamulonis et al., 2011). These physical parameters found in the environment of the microbial mat, along with our results, affirm the necessity of precise phototactic movement as a primary response stimulus of motility.

Similar cyanobacteria found in hot water springs in arctic regions responded primarily to various other stimuli other than light (Castenholz, 1968). These cyanobacteria were motile as a mat community in response to fluctuating stimuli in their environment, such as water temperature due to cold-water runoff mixing with the hot water springs. The fluctuation of the physical parameters of this system proved to dictate the motile behavior of these organisms. However, the environment of ECB and especially the MIS maintain consistency in physical-chemical conditions due to the constant influx of groundwater into the system. This inflow maintains a stable level of dissolved oxygen, water temperature, flow, and conductivity (Ruberg et al., 2008). The amount of light present at the level of the mat is therefore the most variable motility stimulus, reliant upon diurnal light cycling, cloud cover, weather patterns, and water depth clarity. In light of Castenholz’s (1968) description of motile cyanobacteria traveling in response to the most variable stimuli in the environment and our own measurements of the ECB and MIS’s relative physical-chemical constancy, the observation of precise phototactic movement in the lab makes good ecological sense. Due to the similarities between these organisms in the Arctic and Lake Huron, a similar ancestry is possible with subsequent divergent evolution due to environmental stresses.

### Carbon Burial

When small bits of shell were placed upon an intact mat overlaying sediment, the mat eventually grew over them. Over time, such filament overgrowth on falling particles may effectively bury organic detritus from sinking plankton and other organisms of the MIS into the benthic carbon reservoir. Previous research has shown that the majority of organic matter in the carbon sink originated from phytoplankton that has settled from the water column onto the mat (Nold et al., 2013). This organic matter falls through the mat by the movement of *Oscillatoria* into the underlying anaerobic sediments where they become preserved. The scenario works like this: when decaying plankton and other suspended debris fall onto the mat, the cyanobacterial filaments climb over it to seek sunlight and push the detritus down into the anaerobic sediment where it tends to be preserved (Biddanda et al., 2012). The ability of mats to quickly bury objects substantially larger than plankton demonstrates the enhanced capacity of sinkhole ecosystems overlain by cyanobacterial mats to efficiently bury carbon.

Motile mat cyanobacteria may inadvertently facilitate burial of planktonic carbon by continually migrating toward light at the mat’s surface during the day. Indeed, MIS is a carbon reservoir, with 18 m of carbon-rich sediment buried under the microbial mat (∼15% organic carbon) as shown by acoustic sub-bottom profiling and geochemical analysis (Nold et al., 2010, 2013; Biddanda et al., 2012). Not only is the carbon content of these sediments one of the highest recorded, the estimated sediment accrual rate of ∼1–2 m per millennium (if confirmed by further studies) is among the highest for any natural ecosystem. The source of this sedimentary carbon, as determined by its stable isotope signature, appears to be exclusively settling plankton and debris from the overlying lake water column (Sanders et al., 2011; Nold et al., 2013). Once the organic carbon is below the mat in the anaerobic layer, it is slow to degrade and becomes preserved as observed in sulfur-fed springs of Lake Cadagno in Switzerland (Hays, 2006; Hebting et al., 2006). Indeed, the gradual microbial metabolism of this carbon reservoir under low redox conditions creates hydrogen sulfide and methane bubbles which may become trapped in the tightly woven microbial mat, creating finger-like projections of the mat (Biddanda et al., 2009).

## Conclusion

Ubiquitous, but tiny microbes continually obtain and use information from their microenvironment to meet the fundamental challenges of everyday life (Dusenbery, 1996; McBride, 2001). Microbes responding collectively to microenvironmental gradients in their habitats are the engines that drive the cycling of major bioactive elements on Earth (National Academy of Sciences, 2007; Falkowski et al., 2008; Stocker, 2012; Schlesinger and Bernhardt, 2013). Long cyanobacterial filaments that are fast-moving are able to optimize light capture (Tamulonis et al., 2011). Benthic cyanobacterial mats covering submerged sinkhole ecosystems of Lake Huron exhibit rapid motility of their filaments in response to light and other environmental cues. Such integrated filament motility may play critical roles in mat structure, formation, and maintenance of redox gradients, optimizing individual and collective photosynthesis in a light-poor environment and favoring enhanced sedimentary carbon accrual. Thus, our motility-related findings in this study with modern-day cyanobacteria have important ramifications for advancing our understanding of the evolution of the early Earth’s atmosphere, as influenced by similar microbial communities that may have colonized the Proterozoic seas.

Lake Huron sinkholes represent the only known refugia for such cyanobacteria in the Northern hemisphere; ice-covered Antarctic lakes are the only other known habitat. The submerged sinkhole mat communities of Lake Huron thrive in oxygen-poor and sulfur-rich groundwater seeping out of ancient marine evaporites in the region’s karst aquifers, and are likely related to cyanobacteria that first oxygenated the biosphere billions of years ago (Biddanda et al., 2012; Voorhies et al., 2012). Such taxonomically simple but metabolically versatile microbial communities represent a major gap in our understanding of how life shaped the chemistry and habitability of our planet, and makes them ideal systems for exploring important unresolved issues in evolution, biodiversity and physiology. For example, within the submerged sinkhole mat communities of Lake Huron, perhaps the oldest form of biological warfare – the one between cyanobacteria and cyanophage viruses – still rages (Voorhies et al., 2015). Thus, the cyanobacteria-dominated submerged sinkholes are model communities for examining microbial physiology in an extreme environment with potential applications to our search for life beyond the home planet. Despite the uniqueness and fragility of Lake Huron’s sinkhole communities, their geographic distribution and biological diversity is relatively unexplored, and their ecophysiology remains largely understudied. Such knowledge will be critical to the conservation of these small and distantly distributed ecosystems in a rapidly changing world.

Cyanobacteria continue to play important ecological roles in modern Earth (Falkowski et al., 2008). It is of considerable biogeochemical interest that ecologically similar modern-day microbial mat communities that reduce carbon and oxidize sulfur are found in geographically distant locations such as Antarctic lakes, thermal springs, and other extreme sulfur-rich environments that resemble conditions on early Earth. In modern-day low-oxygen refugia such as submerged sinkholes, motility may confer cyanobacteria the ability to thrive under low-light conditions by maximizing their metabolism across sharply varying gradients of redox and chemical resources in their benthic habitats. If such motile traits prevailed in cyanobacteria during the Proterozoic, they might have been critical to the filaments staying on top of the benthos in the turbid shallow ocean – optimizing photosynthesis and burying away planktonic organic carbon production – leading to the eventual oxygenation of the environment (Allwood et al., 2006; Lyons et al., 2014). Just as Leeuwenhoek first visualized the “prettily a-moving” microbes in drops of lake water by peering through his home-made microscope, modern-day low-oxygen, high-sulfur ecosystems that are dominated by motile cyanobacterial mats may provide a “working window” for peering into our distant evolutionary past and enable us to ponder their continuing biogeochemical role in today’s world.

## Author Contributions

BB planned and designed the study, obtained funding, participated in the sampling and the running of experiments, wrote and reviewed the manuscript.

AM conducted the sampling, designed and carried out the experiments, drew up the first outline of the paper, wrote and reviewed the manuscript.

SL conducted sampling, preliminary motility studies, wrote and reviewed the manuscript.

MS assisted with sampling, conducted the photosynthetic efficiency experiments, wrote and reviewed the manuscript.

AW assisted with sampling, conducted preliminary motility experiments, prepared figures, wrote and reviewed the manuscript.

## Conflict of Interest Statement

The authors declare that the research was conducted in the absence of any commercial or financial relationships that could be construed as a potential conflict of interest.
